# Supplementary material for: Costs of services and funding gap of the Bangladesh National Tuberculosis Control Programme 2016–2022: An ingredient based approach
Source: PLoS One. 2023 Jun 2;18(6):e0286560. doi: 10.1371/journal.pone.0286560 (PMC10237497; doi:10.1371/journal.pone.0286560)
Supplement: S1 Table — (DOCX) [file pone.0286560.s001.docx]

S1. List of sites visited for data collection

1. Rajshahi chest disease hospital
2. Rajshahi chest disease clinic
3. Tangail chest disease clinic
4. Rajshahi regional TB reference laboratory (RTRL)
5. National TB reference laboratory (NTRL)
6. National institute of diseases of chest and hospital (NIDCH)
7. Damien’s Mymensingh TB hospital
8. DOTs corner at Kalihati Upazila Health Complex
9. DOTs corner at Ghatail Upazila Health Complex
10. DOTs corner at Modhupur Upazila Health Complex
11. BRAC’s TB diagnostic centre (TDC) at Dhaka and
12. icddr,b TB Screening and Treatment Centre in Dhaka
