# Supplementary material for: Costs of services and funding gap of the Bangladesh National Tuberculosis Control Programme 2016–2022: An ingredient based approach
Source: PLoS One. 2023 Jun 2;18(6):e0286560. doi: 10.1371/journal.pone.0286560 (PMC10237497; doi:10.1371/journal.pone.0286560)
Supplement: S2 Table — (DOCX) [file pone.0286560.s002.docx]

S2. Diagnostic algorithms

| Sl. | Diagnostic algorithms |
| --- | --- |
| 1 | Prolonged cough -> Sputum Smear -> Clinical Diagnosis |
| 2 | Prolonged cough -> CXR -> GeneXpert |
| 3 | Prolonged cough -> GeneXpert |
| 4 | Prolonged cough -> CXR -> Sputum Smear |
