# Supplementary material for: Costs of services and funding gap of the Bangladesh National Tuberculosis Control Programme 2016–2022: An ingredient based approach
Source: PLoS One. 2023 Jun 2;18(6):e0286560. doi: 10.1371/journal.pone.0286560 (PMC10237497; doi:10.1371/journal.pone.0286560)
Supplement: S3 Table — (DOCX) [file pone.0286560.s003.docx]

S3. Sensitivity and specificity of different diagnostics

| **Sensitivity** |  |  |  |  |
| --- | --- | --- | --- | --- |
|  | HIV- |  | HIV+ |  |
|  | SSpos | SSneg | SSpos | SSneg |
| Patient has >2 weeks' cough | 57.8 | 57.8 | 57.8 | 57.8 |
| Patient has cough, any duration | 62.7 | 62.7 | 62.7 | 62.7 |
| Patient has any TB symptom | 77 | 77 | 77 | 77 |
| Chest X-Ray (TB abnormality) | 86.8 | 86.8 | 86.8 | 86.8 |
| Chest X-Ray (any abnormality) | 97.8 | 97.8 | 97.8 | 97.8 |
| Smear microscopy test | 100 | 0 | 100 | 0 |
| GeneXpert test | 98.3 | 79.3 | 98.3 | 71.8 |
| Clinical diagnosis without x-ray | 24 | 24 | 24 | 24 |
| Clinical diagnosis with x-ray | 24 | 24 | 24 | 24 |
| Culture test | 100 | 100 | 100 | 100 |
|  |  |  |  |  |
|  |  |  |  |  |
| **Specificity** |  |  |  |  |
|  | HIV- | HIV+ |  |  |
|  |  |  |  |  |
| Patient has >2 weeks' cough | 94.7 | 94.7 |  |  |
| Patient has cough, any duration | 77.5 | 77.5 |  |  |
| Patient has any TB symptom | 67.7 | 67.7 |  |  |
| Chest X-Ray (TB abnormality) | 89.4 | 89.4 |  |  |
| Chest X-Ray (any abnormality) | 75.4 | 75.4 |  |  |
| Smear microscopy test | 97.4 | 97.4 |  |  |
| GeneXpert test | 99 | 99 |  |  |
| Clinical diagnosis without x-ray | 94 | 94 |  |  |
| Clinical diagnosis with x-ray | 94 | 94 |  |  |
| Culture test | 100 | 100 |  |  |
