# Supplementary material for: Costs of services and funding gap of the Bangladesh National Tuberculosis Control Programme 2016–2022: An ingredient based approach
Source: PLoS One. 2023 Jun 2;18(6):e0286560. doi: 10.1371/journal.pone.0286560 (PMC10237497; doi:10.1371/journal.pone.0286560)
Supplement: S4 Table — (DOCX) [file pone.0286560.s004.docx]

S4. Coverage of diagnostic algorithm in Care and Control of OHT used for modeling

| HIV- |  |  |  |  |  |
| --- | --- | --- | --- | --- | --- |
| Year | Prolonged cough -> Sputum Smear -> Clinical Diagnosis | Prolonged cough -> CXR -> GeneXpert | Prolonged cough -> GeneXpert | Prolonged cough -> CXR -> Sputum Smear | Total |
| 2016 | 95 | 0 | 4 | 1 | 100 |
| 2017 | 86 | 9 | 4 | 1 | 100 |
| 2018 | 70.3 | 20.2 | 7.5 | 2 | 100 |
| 2019 | 64 | 26 | 8 | 2 | 100 |
| 2020 | 52 | 36 | 10 | 2 | 100 |
| 2021 | 41 | 45 | 13 | 1 | 100 |
| 2022 | 19 | 60 | 20 | 1 | 100 |
| HIV+ |  |  |  |  |  |
| 2016 | 95 | 0 | 4 | 1 | 100 |
| 2017 | 86 | 9 | 4 | 1 | 100 |
| 2018 | 70.3 | 20.2 | 7.5 | 2 | 100 |
| 2019 | 64 | 26 | 8 | 2 | 100 |
| 2020 | 52 | 36 | 10 | 2 | 100 |
| 2021 | 41 | 45 | 13 | 1 | 100 |
| 2022 | 19 | 60 | 20 | 1 | 100 |
