# Supplementary material for: Costs of services and funding gap of the Bangladesh National Tuberculosis Control Programme 2016–2022: An ingredient based approach
Source: PLoS One. 2023 Jun 2;18(6):e0286560. doi: 10.1371/journal.pone.0286560 (PMC10237497; doi:10.1371/journal.pone.0286560)
Supplement: S6 Table — (DOCX) [file pone.0286560.s006.docx]

S6. Description of different TB treatment regimen included in the costing

| **Treatment category** | **Regimen** |
| --- | --- |
| Category I: First-line TB treatment: Initial treatment for adult | **Intensive phase: 2 (HRZE)**  **Continuation phase: 4 (HR)**  (6-month treatment regimen composed of Isoniazid, Rifampicin, Pyrazinamide, Ethambutol for the first 2 months and Isoniazid, Rifampicin for 4 months) |
| Treatment for children (smear negative PTB) | **Intensive phase: 2 (HRZ)**  **Continuation phase: 4 (HR)**  (6-month treatment regimen composed of Isoniazid, Rifampicin, Pyrazinamide for the first 2 months and Isoniazid, Rifampicin for 4 months) |
| Category II: First-line TB treatment: Previously treated adult | **Intensive phase: 2 (HRZE) S**  **Continuation phase: 5 (HRE)**  (7-month treatment regimen composed of Isoniazid, Rifampicin, Pyrazinamide, Ethambutol, Streptomycin for the first 2 months and Isoniazid, Rifampicin, Ethambutol for 5 months) |
| Long regimen treatment for MDR TB (20 months)** | **Intensive phase: 8 (Km-Z-Lfx-Eto-Cs)**  **Continuation phase: 12 (Lfx-Eto- Cs-Z)**  (20-month treatment regimen composed of Kanamycin for the first 8 months and levofloxacin, Ethionamide, Cycloserine, Pyrazinamide for 20 months) |
| Short regimen treatment for MDR-TB (9 months)** | **Intensive phase: 4 (Km-Mfx-Pto-Cfz-E-H-Z)**  **Continuation phase: 5 (Cfz-E-H-Z)**  (9-month treatment regimen composed of Kanamycin, Moxifloxacin, Prothionamide for the first 4 months and Moxifloxacin, Ethambutol, Isoniazid, Pyrazinamide for 9 months) |
| XDR TB treatment regimen (24 months)** | **Intensive phase: 12(Cm-Z-Mfx-PAS-Cs-Amx/Clv- Lzd -Cfz)**  **Continuation phase: 12(Z-Mfx-PAS-Cs-Amx/Clv- Lzd -Cfz)**  (24-month treatment regimen composed of Capreomycin for the first 12 months and Pyrazinamide, Moxiflixacin, PAS, Cycloserine, Amoxicilin, Lenozolid, Clofazimine for 24 months) |
| **Prevention** |  |
| IPT for children under 5 years of age | **6 H**  (6-month treatment regimen composed of Isoniazid for 6 months) |
| **New treatment for children (started from 2019)** |  |
| 3 HP for children 2 to 11 years old | **3 HP**  (3 months treatment regimen composed of Isoniazid and Rifapentine for 3 months- once weekly) |
| IPT for children <2 years of age | **6 H**  (6-month treatment regimen composed of Isoniazid for 6 months) |
| New regimen for child retreatment cases (Pulmonary negative) | **6 (HRZ)-E**  (6-month treatment regimen composed of Isoniazid, Rifampicin, Pyrazinamide and Ethambutol for 6 months) |
| New child retreatment cases- Meningitis, bone, Neurological TB | **12 (HRZ)-E-Lfx**  (12-month treatment regimen composed of Isoniazid, Rifampicin, Pyrazinamide, Ethambutol and Levofloxacin for 12 months) |
| Levofloxacin based regimen for child retreatment cases (Pulmonary positive) | **6 Lfx-(HRZ)-E**  (6-month treatment regimen composed of Levofloxacin, Isoniazid, Rifampicin, Pyrazinamide and Ethambutol for 6 months) |
| **New treatment for adults (started from 2019)** |  |
| New Levofloxacin based regimen for Adult retreatment cases (Pulmonary positive/Extrapulmonary TB) | **6 (H)REZ- Lfx**  (6-month treatment regimen composed of Isoniazid, Rifampicin, Pyrazinamide, Ethambutol and Levofloxacin for 6 months) |
| **New treatment for MDR TB (started from 2019)** |  |
| New regimen for adult retreatment cases (Pulmonary negative) | **6 HRZE**  (6-month treatment regimen composed of Isoniazid, Rifampicin, Pyrazinamide and Ethambutol for 6 months) |
| New regimen for adult retreatment cases- Meningitis, bone, and Neurological TB | **12 HRZE-Lfx**  (12-month treatment regimen composed of Isoniazid, Rifampicin, Pyrazinamide, Ethambutol and Levofloxacin for 12 months) |
| New MDR shorter regimen (9 months) | **Intensive phase: (4 -6) Bdq (6m or longer)-Lfx-Pto/Eto-Cfz-Z-H high dose-E**  **Continuation phase: 5 Lfx-Cfz-Z-E**  (9 months treatment regimen composed of Bedaquiline for first 6 months; Prothionamide, high dose Isoniazid, Ethambutol for first 4 months; Levofloxacin, Clofazimine, Pyrazinamide, Ethambutol for 9 months) |
| New MDR longer regimen (20 months) | **20 Bdq(6m)-Lfx-Lzd-Cfz-Z**  (20-month treatment regimen composed of Bedaquiline for the first 6 months and Levofloxacin, Linezolid, Clofazimine, Cycloserine and Pyrazinamide for 20 months) |
| New MDR with additional resistance (6 months Bedaquiline regimen) | **6 Bdq-Pa-Lzd (BPaL regimen)**  (6-month treatment regimen composed of Bedaquiline, Pretomanid and Linezolid for 6 months) |
