# Supplementary material for: Costs of services and funding gap of the Bangladesh National Tuberculosis Control Programme 2016–2022: An ingredient based approach
Source: PLoS One. 2023 Jun 2;18(6):e0286560. doi: 10.1371/journal.pone.0286560 (PMC10237497; doi:10.1371/journal.pone.0286560)
Supplement: S7 Table — (DOCX) [file pone.0286560.s007.docx]

**S7 List of anti-TB drugs**

| ***First-line oral anti-TB drugs*** |
| --- |
| - Isoniazid (H) - Rifampicin (R) - Ethambutol (E) - Pyrazinamide (Z) - Rifapentin (Rpt) |
| ***Second-line injectable anti-TB drugs*** |
| *Fluroquinolones:*   - Levofloxacin (Lfx) - Moxifloxacin (Mfx)   *Injectable:*   - Streptomycin (S) - Kanamycin (km) - Amikacin (Am) - Capreomycin (Cm)   *Other core second-line drugs:*   - Ethionamide (Eto) - Prothionamide (Pto) - Cycloserine/terizidone (Cs) - Linezolid (Lnz) - Clofazimine (Cfz)   *Add-on agents:*   - Amoxicillin (Amx) - Delamanid (Dlm) - Bedaquiline (Bdq) - p-Aminosalicylic acid (PAS) |
| ***Others*** |
| - Pyridoxine |
