# Supplementary material for: Costs of services and funding gap of the Bangladesh National Tuberculosis Control Programme 2016–2022: An ingredient based approach
Source: PLoS One. 2023 Jun 2;18(6):e0286560. doi: 10.1371/journal.pone.0286560 (PMC10237497; doi:10.1371/journal.pone.0286560)
Supplement: S8 Table — (DOCX) [file pone.0286560.s008.docx]

**S8 List of supplies/ reagents**

| FM-LED 700-smears |
| --- |
| Z-N-700-smears |
| Sputum cup |
| N-95 mask |
| Reagent kit (Cartidge + Reagent + dropper) |
| 75% ethanol/Hexisol, |
| Immersion oil |
| Lens tissue (paper) |
| Tissue paper |
| 5% phenol |
| Falcon tube |
| Gloves |
| Biohazard bag |
| Marker pen |
| Absorbent paper |
| Cetyl pyridine chloride (CPC) NaCl solution |
| 4% NaOH solution |
| Phosphate buffer solution |
| Culture media |
| Disposable pasteur pipettes, graduated, non-sterile, 155 mm, 3 ml |
| Microscope slide, lime-soda-glass |
| INH powder |
| Rifampicin powder |
| Dihydro Streptomycin(DS) powder |
| Ethambutol (E) powder |
| P- nitro benzoic acid (PNB) powder |
| Sterile 0.01% Tween 80® |
| MGIT 960 growth supplement kit (OADC+PANTA) |
| BACTEC™ MGIT™ 960 SIRE kit, One kit is sufficient for 40 test |
| BBL MGIT Tubes for use in Bactec MGIT 960 (7ml) |
| BACTEC™ MGIT™ PZA Tubes |
| BBL middle brook 7H9 Broth with glycerol 5ml |
| BBl MGIT Tube for Manual method (4ml) |
| MGIT 960 PZA kit |
| Disposable loops 10 µl |
| Distilled water |
| Kanamycin lyophilized drug, 830 µg/ vial |
| Moxifloxacin lyophilized drug, 498 µg /vial |
| Amikacin lyophilized drug, 332 µg/ vial |
| Capreomycin lyophilized drug, 830 µg/ vial |
| Ofloxacin lyophilized drug, 664 µg / vial |
