# Supplementary material for: Costs of services and funding gap of the Bangladesh National Tuberculosis Control Programme 2016–2022: An ingredient based approach
Source: PLoS One. 2023 Jun 2;18(6):e0286560. doi: 10.1371/journal.pone.0286560 (PMC10237497; doi:10.1371/journal.pone.0286560)
Supplement: S9 Table — (DOCX) [file pone.0286560.s009.docx]

**S9 Cost of constructing new facilities by type of assumed units in 2016 US$.**

| **Types of facility** | **Cost of building** | **Cost of equipment** | **Cost of furniture** |
| --- | --- | --- | --- |
| DOTs Centre | 7,744.2 | 49.2 | 407.0 |
| Microscopy / Peripheral Microscopy Centre | 5,632.1 | 1,163.7 | 1,125.7 |
| X-pert Centre | 4,928.1 | 42,547.7 | 984.3 |
| TB Clinic | 114,438.2 | 103.9 | 4,597.1 |
| TB Hospital | 719,155.1 | 42,697.7 | 118,740.0 |
| X-ray Centre | 7,392.2 | 19,740.9 | 237.3 |
| Culture & DST Centre | 105,602.8 | 136,931.3 | 13,421.4 |
