# Supplementary material for: Costs of services and funding gap of the Bangladesh National Tuberculosis Control Programme 2016–2022: An ingredient based approach
Source: PLoS One. 2023 Jun 2;18(6):e0286560. doi: 10.1371/journal.pone.0286560 (PMC10237497; doi:10.1371/journal.pone.0286560)
Supplement: Attachment — Submitted filename: Response to reviewers.docx [file pone.0286560.s012.docx]

**Responses to reviewer’s comments**

**Reviewer 1, Comment 3:** I feel this was not properly explained in response and could be made clearer in the text with diagnostics and treatments referenced. Box 1 would benefit from some separation for tests/treatments specific to TB infection, drug-susceptible TB disease, and drug-resistant TB disease.

**Response:** Many thanks for the comment. We have added the following lines as follows “The list of interventions (investigations and treatments) considered for costing is detailed in Box 1. The interventions included several tests for TB diagnosis namely, Microscopy, X-ray, Culture, Drugs susceptibility testing (DST), GeneXpert, LPA (Line Probe Assay), FNAC (Fine Needle Aspiration Cytology), and Mendel–Mantoux test [12]. Additionally, few supplementary tests are done for the baseline and follow-up of MDR-TB treatment, such as Audiometry, ECG, Complete Blood Count, Serum Creatinine, Serum Electrolyte, Serum Bilirubin, Alkaline Phosphatase, Thyroid Function Test (TSH) [13]. The interventions related to treatment include ‘preventive therapy’ [14], treatment for ‘drug susceptible TB’ patients [12], and ‘drug-resistant TB’ patients [13]. ” We have now updated the box as per suggestion. ***Please see page 5, line 121-128***

**Reviewer 1, Comment 4; Reviewer 2:** The authors did not address this comment adequately. The question had to do with selection of centers and the description is more confusing that before. I am not entirely clear on the procedures.

**Response**: We apology for the confusion. We have now revised the section as follows “We collected facility-related data from twelve purposively selected facilities representing different levels of diagnosis and treatment offered by the government and NGOs service providers. The facilities selected were from four main types of providers: 1) DOTs Centres (government and NGO), 2) chest disease clinics (CDC), 3) chest disease hospitals (CDH) (government and NGO), and 4) national and regional TB reference laboratories (Supplementary Table 1). These facilities were selected in consultation with the NTP as representative of facilities providing TB care in Bangladesh.” ***Please see page 4, line 99-105***

**Reviewer 1, Comment 8:** I think sticking to USD in all tables is advised.

**Response**: We have now USD in all tables.

**Reviewer 2, Comment 1:** It is still unclear what happened in the years where a funding gap existed. I think this comes back to using modeled estimates of resource needs and looking at what actually was done. The discussion would benefit from this information: what were the consequences of underfunding?
**Response**: Many thanks for this query. It is evident that although the tuberculosis services are free, limited access to quality services and lack of screening facilities are major barriers in adequate detection and treatment of the disease. Funding gap for TB programme may increase these challenges. We have added the following lines in the discussion.

“It is evident that although the TB services are free in Bangladesh, access to quality services and modern screening facilities are major challenges in adequate detection and treatment of TB cases [31]. Funding gap may enhance such challenges, increase catastrophic expenditure [32], and decrease case notification as happened in 2011 due to the funding gap from GFATM [33]. However, we did not study the consequences of underfunding during 2019-2022. It would be interesting to assess the consequences of the funding gap in future studies.” ***Please see page 14, line 323-327***

**Other comments:**
Line 67-72 -- Perhaps simply refer to Global Health Cost Consortium (<https://ghcosting.org/>), which summarizes the available cost data for tuberculosis. You can also look to Value TB studies. The current summation is inadequate.

**Response:** Many thanks for this suggestion. We have now added the reference.

Lines 93-94 - unclear language "We collected facility-related data from purposively selected four types of twelve facilities representing different level of diagnosis and treatment of government and NGOs"

**Response**: We have now revised the lines as follows “We collected facility-related data from twelve purposively selected facilities representing different levels of diagnosis and treatment offered by the government and NGOs service providers. The facilities selected were from four main types of providers: 1) DOTs Centres (government and NGO), 2) chest disease clinics (CDC), 3) chest disease hospitals (CDH) (government and NGO), and 4) national and regional TB reference laboratories” ***Please see page 4***

**Results and Discussion** -- these should be separate.

**Response**: We followed the journal suggested heading for formatting.
